# Atmospheric Flame Vapor Deposition of 1D and 2D Nanostructured Vanadium Pentoxide on Diverse Substrates

**DOI:** 10.3390/nano15100709

**Published:** 2025-05-08

**Authors:** Kai Zhou, Lili Cai

**Affiliations:** 1 Department of Mechanical Science and Engineering, The Grainger College of Engineering, University of Illinois Urbana-Champaign, Urbana, IL 61801, USA; kaizhou2@illinois.edu; 2 Frederick Seitz Materials Research Laboratory, The Grainger College of Engineering, University of Illinois Urbana-Champaign, Urbana, IL 61801, USA

**Keywords:** flame synthesis, flame vapor deposition, vanadium oxide synthesis, vanadium pentoxide, V_2_O_5_

## Abstract

Vanadium pentoxide (V_2_O_5_) has attracted considerable interest owing to its unique chemical and physical properties. However, traditional synthesis methods are often time-consuming, complex, and difficult to scale, limiting the broader applications of V_2_O_5_. Herein, we present a flame vapor deposition (FVD) method to enable rapid, scalable, and one-step synthesis of various V_2_O_5_ nanostructures under ambient pressure conditions. By optimizing critical synthesis parameters, specifically, source temperature (840 °C) and substrate temperature (610 °C), we achieved highly crystalline, one-dimensional (1D) V_2_O_5_ nanorods on a variety of substrates, including silicon (Si), fluorine tin doped (FTO) glass, stainless steel, and silicon dioxide (SiO_2_). Moreover, we demonstrate the rapid growth of ultrathin, two-dimensional (2D) V_2_O_5_ nanoflakes with nanometer-scale thickness, as well as enhanced uniformity and coverage density with an externally applied electric field. This FVD method provides a simple, efficient, and scalable approach for synthesizing advanced V_2_O_5_ nanostructures, significantly expanding opportunities for their integration into various technological applications.

## 1. Introduction

Vanadium exhibits a broad spectrum of oxidation states from +2 to +5, leading to diverse structures and unique functional properties. Among these oxides, vanadium pentoxide (V_2_O_5_) has received significant attention, due to its unique layered structure, wide bandgap (2.3 eV), excellent electrochromic properties, and notable thermal and chemical stability stemming from its highest oxidation state (+5). Its distinctive layered structure, characterized by strong in-plane covalent bonding and weak interlayer van der Waals forces, facilitates efficient ion intercalation. These features make V_2_O_5_ particularly attractive for various applications, including gas sensing [1,2,3], catalysis [4,5,6], electrochromic devices [7,8,9], batteries [10,11,12,13], and electrochemical capacitors [14,15,16].

To date, several methods have been explored to synthesize nanostructured V_2_O_5_, including hydrothermal synthesis [10,17,18,19,20], precipitation [21,22,23,24], thermal evaporation [25,26,27,28], sputtering [29,30,31], hot filament chemical vapor deposition (HFCVD) [3,32], and sol–gel synthesis [8,33,34,35,36,37]. Despite their effectiveness, these techniques often involve extended processing times, complex experimental setups, or stringent vacuum or low-pressure conditions, limiting scalability and practical applications.

Flame synthesis, in contrast, is an industrially relevant combustion-based method recognized for scalability, cost-effectiveness, and rapid throughput. It has been widely implemented for industrial nanoparticle production, with a market exceeding USD 15 billion annually and an output of millions of tons [38]. Flame synthesis has also been successfully adopted at the lab scale to fabricate multiple transitional metal oxides in high-aspect-ratio nanostructure forms (e.g., nanowires, nanoplates, nanorods, etc.), including WO_3_ [39], TiO_2_ [40,41,42], and MnO_3_ [43]. However, despite the demonstrated advantages, the flame-based synthesis of vanadium oxides, particularly in aligned one-dimensional (1D) and ultrathin two-dimensional (2D) nanostructures, remains relatively underexplored. In this regard, previous flame synthesis studies often faced significant limitations, including challenges in achieving precise control over the growth morphology, uniformity, and quality.

In this work, we present an atmospheric-pressure flame vapor deposition (FVD) method to synthesize nanostructured vanadium oxide from 1D to 2D on diverse substrates using a methane–air flat flame. This FVD technique leverages the post-flame gaseous conditions, with precisely controlled equivalent ratios (φ) for fuel-lean (oxygen-rich) environments, to vaporize and oxidize metallic vanadium precursors without complex reactor setups or low-pressure constraints. We systematically investigated the key process parameters, such as oxidation temperature, substrate temperature, substrate type and electric bias, elucidating their roles in the growth of V_2_O_5_ nanostructures. Notably, we not only synthesized densely pack, vertically aligned V_2_O_5_ nanorods, but also achieved the growth of ultrathin 2D V_2_O_5_ nanoflakes, demonstrating precise morphological control previously unattainable in ambient-pressure flame-based processes. This advancement significantly expands the possibilities for scalable production of dimensionally controlled V_2_O_5_ nanostructures and open new avenues for their integration into functional devices, including batteries, electrochromics, sensors, and optoelectronics.

## 2. Methods

### 2.1. Substrate Pretreatment

Si and SiO_2_ (300 nm)/Si wafers were acquired from University Wafer (Boston, MA, USA). Fluorine-doped tin oxide (FTO) glass (2.2 mm thick) was obtained from MSE Supplies (Tucson, AZ, USA). Stainless-steel foil (430) with a thickness of 0.508 mm was purchased from McMaster Carr (Elmhurst, IL, USA). All substrates (~2 × 1 cm) were cleaned sequentially via sonication in acetone, isopropanol (IPA), and deionized (DI) water, each for 10 min. For ultrathin 2D flake growth, the cleaned substrates were dried by air blow and used immediately without further treatment. For 1D nanostructure growth, the substrates underwent an additional seeding step. The seeding solution was prepared by dissolving 0.0136 g vanadyl acetylacetonate (VO(acac)_2_, Sigma Aldrich, St. Louis, MO, USA) in 5 mL ethanol, then sonicating for 10 min to form a 0.01 M solution. Each substrate was coated with 0.5 mL of the seeding solution using a spin coater (Laurell Technologies, Lansdale, PA, USA) at 500 RPM for 5 s and 3000 RPM for 25 s. After coating, the coated substrates were left in air to dry naturally.

### 2.2. Flame Synthesis

**Figure 1:**
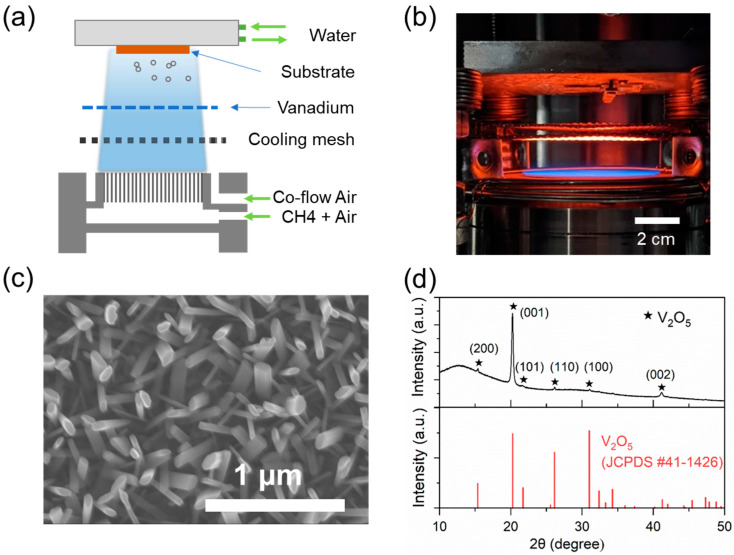
(**a**) Schematic and (**b**) photograph of the flame vapor deposition setup. (**c**) SEM image of V_2_O_5_ nanofibers grown on a Si substrate. (**d**) XRD pattern confirming the orthorhombic crystal structure of the as-grown V_2_O_5_ (JCPDS card no. 41-1426, orthorhombic).

Flame vapor deposition was performed using a McKenna-type burner (6 cm diameter, Holthuis and Associates, Sebastopol, CA, USA) at atmospheric pressure (Figure 1a). The burner has a porous sintered plug in the center, where a stable, radially uniform flame is generated. Methane (CH_4_) served as the flame fuel, and air was used as the oxidizer. Gas flow rates of CH_4_ and air were precisely controlled using mass flow controllers (Alicat Scientific, Tucson, AZ, USA), in which CH_4_ was fixed at 2.00 standard liters per minute (SLPM), while the air was adjusted according to the targeted equivalence ratio (φ). For example, the air flow rate was set to 21.16 SLPM for an equivalence ratio of φ = 0.9. To enhance flame stability, an extra air co-flow (100 SLPM) was also introduced from the annular shroud surrounding the burner center.

During the synthesis, vanadium wires (0.25 mm diameter) were positioned downstream of the flame and upstream of the growth substrate. The high-temperature, oxygen-rich flame vaporized the vanadium wires, subsequently transporting vanadium oxide vapor towards the substrate where growth occurred. The source and substrate temperatures were monitored using K-type thermocouples (Omega Engineering, Norwalk, CT, USA). These temperatures were controlled by employing a stainless-steel wire mesh (McMaster-Carr), which provided passive cooling effects [39] when placed upstream of the vanadium source, and a water-cooled plate holder with adjustable temperature control through cooling water.

### 2.3. Material Characterization

Scanning electron microscopy (SEM) was performed using a Hitachi S4800 high-resolution microscope (Hitachi Ltd., Tokyo, Japan) to examine the morphology of V_2_O_5_ under different growth temperatures. X-ray diffraction (XRD) was conducted using a Bruker D8 Advance instrument (Bruker Crop., Billerica, MA, USA) with copper K_α_ radiation (λ = 1.54 Å) to analyze the crystal structure of the grown nanomaterials. Atomic force microscopy (AFM, Asylum Research Cypher, Oxford Instruments, Abingdon, UK) was used to measure the nanometer-scale thickness of V_2_O_5_ flakes. Raman spectroscopy (LabRAM HR, Horiba, Kyoto, Japan; 532 nm laser) was utilized to identify and evaluate the oxidation states of the grown nanomaterials. X-ray photoelectron spectroscopy (XPS) was conducted using a Kratos Axis Supra Photoelectron spectrometer (Kratos Analytical, Manchester, UK) to measure the atomic composition at the surface of the 2D growth.

### 2.4. Vanadium Oxide Reduction

Vanadium oxide reduction was conducted in a hydrogen vacuum furnace at 80 mTorr for 1 h in a gas mixture of 5% hydrogen and 50% argon. The furnace, containing the as-grown vanadium oxide, was first degassed to 80 mTorr. Argon and hydrogen were then introduced at flow rates of 950 SCCM and 50 SCCM, respectively. The temperature was ramped from room temperature to 550 °C, held for 1 h, and allowed to cool naturally. Gas flows were stopped before the cooling stage.

## 3. Results and Discussion

### 3.1. FVD Growth of V_2_O_5_

Figure 1 shows a schematic and photograph of the FVD process. Pre-mixed CH_4_ and air are supplied to the porous burner core, producing a radially uniform flame at the burner top surface. The flame operates under fuel-lean conditions (φ < 1) to ensure an oxygen-rich environment for effective oxidation of vanadium metal wire source. The heat and oxygen radicals in the flame vaporize and oxidize vanadium wires to generate vapors, flowing downstream to the pretreated substrate (2 × 1 cm) where deposition occurs. As observed under SEM (Figure 1c), the grown structure on the Si substrate exhibits vertical, quasi-aligned nanorods, with diameters ranging from 50 to 100 nm. Their growth rate was estimated to be around 1–2 μm/h, based on the cross-sectional SEM image. This nanostructure demonstrates high crystallinity corresponding to orthorhombic V_2_O_5_, as confirmed by XRD patterns shown in Figure 1d, matching the reported V_2_O_5_ structure (JCPDS card no. 41-1426, a = 11.516 Å, b = 3.5656 Å, c = 4.3727 Å). In addition, the prominent (001) and (002) diffraction peaks at 2θ= 20.26° and 41.26° indicate that crystal growth predominantly occurs along the [001] layer-stacking direction.

**Figure 2:**
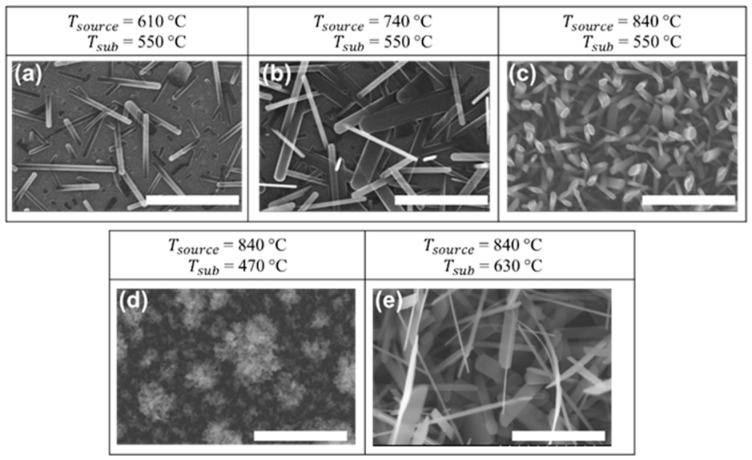
SEM images showing nanostructure morphologies at different growth temperatures. (**a**) T_sub_ = 550 °C, T_source_ = 610 °C; (**b**) T_sub_ = 550 °C, T_source_ = 740 °C; (**c**) T_sub_ = 550 °C, T_source_ = 840 °C; (**d**) T_sub_ = 470 °C, T_source_ = 840 °C; and (**e**) T_sub_ = 630 °C, T_source_ = 840 °C. Conditions in (**a**–**c**) examine the influence of T_source_, while (**c**–**e**) explore the effect of T_sub_. All growth experiments were conducted on Si substrates for 30 min at an equivalent ratio of φ = 0.8. Scalebars: 1 μm.

The morphology of flame-synthesized V_2_O_5_ is primarily governed by two interconnected temperature-dependent mechanisms: source temperature (T_source_) and substrate temperature (T_sub_). Higher T_source_ (≥740 °C) enhances precursor vaporization and oxidation rates, increasing vanadium oxide vapor concentration. This elevated vapor flux intensifies precursor supply to the substrate, driving the formation of denser nanostructures (Figure 2a–c). Conversely, lower T_source_ reduces vapor availability, yielding sparser growth.

On the other hand, substrate temperature (T_sub_) critically affects the growth morphology via nucleation and growth dynamics [44]. A critical T_sub_ threshold of approximately 550 °C was identified, below which amorphous, spherical aggregates resembling “furry balls” form rather than 1D nanorods (Figure 2d). This phenomenon can be attributed to insufficient thermal energy at lower T_sub_, leading to physical condensation and aggregation of vanadium oxide vapor [24]. In contrast, at T_sub_ ≥ 550 °C (Figure 2a–c,e), sufficient thermal energy activates surface diffusion and attachment [44], allowing crystalline 1D nanorod formation, with growth density progressively rising alongside elevated T_source_.

The synergy of T_source_ and T_sub_ determines the final morphology: high T_source_ ensures sufficient vapor concentration, while intermediate T_sub_ balances nucleation density and growth kinetics. At higher T_source_ and higher T_sub_ (Figure 2e), it results in denser nanostructures comprising larger nanorods at the base and thinner, horizontally oriented nanofibers near the surface. This unique morphology likely arises from a two-stage nucleation process: initial nucleation on the substrate surface produces large fibers at the base, followed by secondary nucleation on these initial fibers, resulting in the formation of finer surface nanofibers. Similar temperature-dependent growth behavior has been reported in flame synthesis of tungsten oxide [39]. These results indicate that the formation of dense, crystalline nanostructures requires a high T_source_ (≥740 °C) to ensure sufficient vapor concentration, combined with an optimal T_sub_ between 550 and 630 °C to balance nucleation and growth dynamics.

### 3.2. FVD Growth of V_2_O_5_ on Diverse Substrates

**Figure 3:**
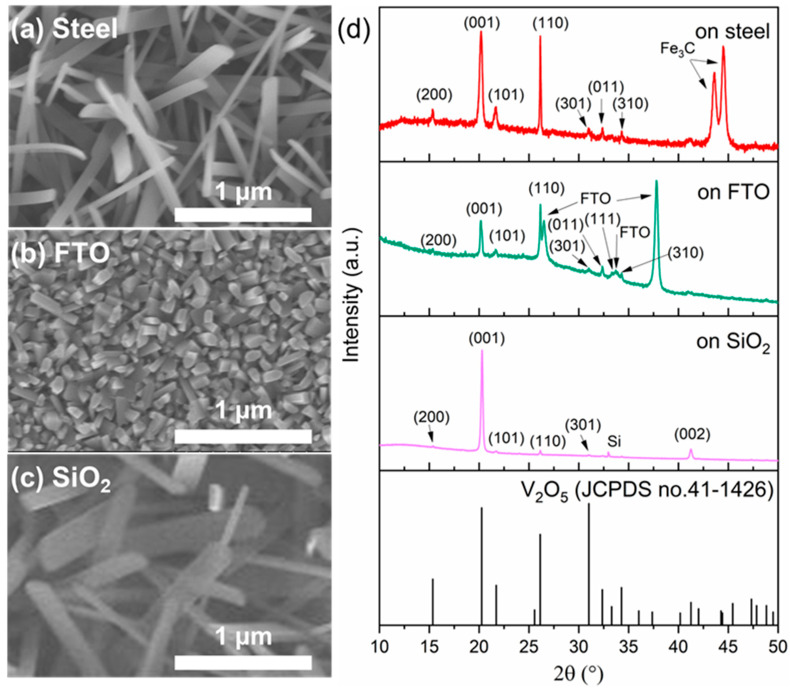
SEM images of the as-grown nanostructures on (**a**) stainless steel, (**b**) FTO glass, and (**c**) SiO_2_/Si substate. (**d**) XRD spectra of the as-grown V_2_O_5_ and the standard orthorhombic V_2_O_5_ reference pattern from JCPDS database (JCPDS no.41-1426). Orthorhombic V_2_O_5_ growth was confirmed on all three substrates. Fe3C peaks match JCPDS no. 35-0772. FTO peaks match JCPDS no.41-1445. All growth experiments here were prepared at T_source_ = 840 °C, T_sub_ = 610 °C, and φ = 0.9 for 30 min.

We further demonstrated that the flame synthesis method for V_2_O_5_ nanostructures can be applied to diverse substrates, including Si, stainless steel, FTO glass, and 300 nm-thick thermally grown SiO_2_ on Si. SEM images (Figure 3a–c) reveal that stainless steel and SiO_2_ substrates yield nanostructures similar in dimension to those on Si substrate (Figure 2c), albeit with a more horizontal orientation. This orientation difference can be attributed to substrate-dependent variations in surface energy, interfacial interactions, and the initial nucleation kinetics. Specifically, the stainless-steel foil substrate exhibits highly polished, polycrystalline surface grains, which can promote horizontal growth of nanostructures with lateral alignment. Similarly, the amorphous nature of SiO_2_ surface may offer a uniformly lower-energy surface without pronounced crystallographic directions, reducing directional preference for vertical growth and thereby enabling more horizontal stacking of nanorods during growth.

In contrast, the nanostructures on FTO substrate are composed of denser and shorter nanorods (Figure 3b). This morphological difference likely arises from the rough, faceted surface structure and high-density surface defects characteristic of FTO glass. Such surface features significantly enhance nucleation efficiency by providing abundant nucleation sites for precursor attachment. The resultant high nucleation density facilitates competitive growth dynamics among closely spaced nuclei, thereby leading to more densely packed, shorter nanorods.

Despite morphological differences among substrates, XRD analysis (Figure 3d) confirmed consistently high crystallinity across all samples, with patterns matching well with the orthorhombic V_2_O_5_ phase (JCPDS no. 41-1426). The prominent peak at 2θ = 20.26° suggests that the as-grown V_2_O_5_ structures exhibit strong preferential orientation along the [001] crystallographic direction. These results, alongside XRD evidence in Figure 1d, convincingly confirm that orthorhombic V_2_O_5_ grows uniformly across all tested substrates. This ability to synthesize V_2_O_5_ on diverse substrates underscores the practicality and versatility of flame synthesis method. For instance, direct growth on conductive substrates like stainless steel and FTO glass enables one-step fabrication of electrochromic devices and functional electrodes, offering substantial advantages over conventional multi-step fabrication processes.

### 3.3. Two-Dimensional V_2_O_5_ Growth

**Figure 4:**
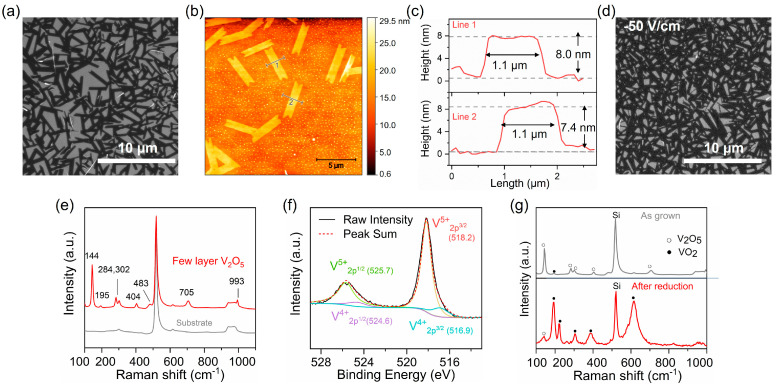
(**a**) Scanning electron microscopy (SEM) image of the ultrathin V_2_O_5_ flake on a SiO_2_ (300nm)/Si substrate. (**b**) Atomic force microscopy (AFM) image and (**c**) corresponding height profiles of the V_2_O_5_ flakes on a SiO_2_/Si substrate. The line 1 and 2 in (**b**) corresponds to the height profile in (c). (**d**) SEM images showing the ultrathin V_2_O_5_ growth in the substrate center edge under electric fields of −50 V/cm. (**e**) Raman spectrum and (**f**) XPS spectrum of the as-grown V_2_O_5_ flake on a SiO_2_/Si substrate. Raman spectrum shows peaks at 144, 195, 284, 302, 404, 483, 705, and 993 cm^−1^, aligning with reported V_2_O_5_ phase [45]. (**g**) Raman spectra of the sample before and after reduction treatment. All growth experiments here were conducted at T_source_ = 840 °C, T_sub_ = 610 °C, and φ = 0.9 for 2 min.

Leveraging the layered van der Waals structure of V_2_O_5_, we successfully achieved the growth of ultrathin 2D V_2_O_5_ flakes comprising only a few layers on a SiO_2_ substrate, as shown in Figure 4. The flakes have lateral dimensions of approximately 4 × 1 μm, as characterized using SEM. AFM height profile measurement confirmed a thickness of around 8 nm (Figure 4a–c). We also investigated the effect of electric fields on flame synthesis of V_2_O_5_. During the FVD process, a DC electric field (−50 V/cm) was applied between the burner (positive terminal) and the substrate (negative terminal). As illustrated in Figure 4d, it was found that applying an electric bias effectively reduces the vertical growth, promoting the formation of more uniform 2D morphology with higher flake density. Raman spectroscopy analysis of the flakes (Figure 4e) exhibits characteristic peaks at 144, 195, 284, 302, 404, 483, 705 and 993 cm^−1^ (resolution of 4 cm^−1^), consistent with the Raman signatures of orthorhombic V_2_O_5_ [1,45,46,47]. Notably, the predominant peak at 144 cm^−1^, corresponding to skeleton bending vibrations, and the 994 cm^−1^ peak, arising from vibrations of the V atom within the V=O double bond, collectively confirm the layered structure and crystalline quality of the flake growth [48].

XPS results (Figure 4f) further confirmed the presence of V_2_O_5_, displaying major peaks at 518.2 eV and 525.7 eV corresponding to 2p3/2 and 2p1/2 peaks of V^5+^, respectively. Additionally, weaker peaks at 516.9 eV and 524.6 eV associated with V^4+^ were detected, indicating formation of oxygen vacancies [49,50]. These V^4+^ states likely result from the partial reduction of vanadium at elevated temperatures (above 200 °C), suggesting the potential conversion of V_2_O_5_ to VO_2_, a material with broad applications, such as radiative cooling [51,52], thermal camouflage [53], and electrochromics [54]. To explore this possibility, the as-grown V_2_O_5_ 2D flake sample was reduced in a hydrogen/argon gas environment at 550 °C for 1 h (see Section 2). Raman spectroscopy (Figure 4g) confirmed the successful transformation into VO_2_, with characteristic peaks at 190, 220, 308, 388, and 616 cm^−1^ [55]. These results highlight the effectiveness of FVD method as a rapid and scalable route for fabricating vanadium oxides in well-controlled nanostructures and variable oxidation states for diverse applications.

## 4. Conclusions

In conclusion, we have demonstrated the effective synthesis of 1D nanorods and 2D nanoflakes of V_2_O_5_ under ambient conditions using a premixed CH_4_/air flat flame. High-quality crystalline orthorhombic V_2_O_5_ nanorods were grown on Si substrate under a fuel-lean flame condition (φ = 0.9). Key synthesis parameters, including T_source_ and T_sub_, were systematically investigated, revealing that T_source_ directly governs the precursor vaporization rate and thus growth rate, while T_sub_ has critical influence on nucleation dynamics. Optimal growth of crystalline nanorod was achieved at T_source_ = 840 °C and T_sub_ = 610 °C. The versatility of this flame synthesis method was demonstrated on diverse substrates, including stainless steel, FTO glass, and SiO_2_, broadening its applicability. Furthermore, ultrathin 2D V_2_O_5_ flakes at a thickness of ~8 nm were successfully fabricated, and their uniformity and density were further enhanced through the application of an external DC electric field. With the demonstrated scalability, adaptability, and simplicity, this work provides a promising approach for producing V_2_O_5_ nanostructures tailored in batteries, electrochromics, sensors, and optics applications.

## Data Availability

The original contributions presented in this study are included in the article. Further inquiries can be directed to the corresponding author.
